# Potential involvement of a TLR4-associated CD155/TIGIT pathway in *Porphyromonas gingivalis*-induced immunosuppressive microenvironment formation in oral squamous cell carcinoma

**DOI:** 10.3389/fonc.2026.1773440

**Published:** 2026-09-30

**Authors:** Xiao Tian, Liming Xu, Yulian Fu, Qiang Qiang, Xue Cao, Jie Wang, Langqing Liu, Bin Ling

**Affiliations:** 1 Department of Oral and Maxillofacial Oncology Surgery, First Affiliated Hospital of Xinjiang Medical University, Urumqi, China; 2 Affiliated Stomatology Hospital, Institute of Stomatology, Xinjiang Uygur Autonomous Region, Urumqi, China

**Keywords:** CD155, oral squamous cell carcinoma, *Porphyromonas gingivalis*, TIGIT, TLR4, tumor immune microenvironment

## Abstract

**Objective:**

Accumulating evidence indicates that oral microbiota dysbiosis and periodontal pathogens contribute to the initiation and progression of oral squamous cell carcinoma (OSCC). However, the molecular mechanisms underlying the interaction between *Porphyromonas gingivalis* and the tumor immune microenvironment remain incompletely understood. This study aimed to investigate the potential role of a TLR4-associated ERK/CD155/TIGIT signaling pathway in *P. gingivalis*-mediated immunosuppressive microenvironment formation during OSCC progression.

**Methods:**

Genes associated with *P. gingivalis* and TIGIT were obtained from GeneCards and The Cancer Genome Atlas (TCGA) databases. Candidate hub genes were identified through bioinformatic analyses. An SCC7 syngeneic orthotopic transplantation mouse model was established, and SCC7 cells were injected together with *Porphyromonas gingivalis*. Tumor growth was assessed by measuring tumor volume and weight. Expression levels of TLR4, ERK1/2, CD155, and TIGIT were evaluated by immunohistochemistry and quantitative real-time PCR (qRT-PCR). Flow cytometry was performed to analyze tumor-infiltrating immune-cell populations. The TLR4 inhibitor TAK-242 was used to investigate the involvement of TLR4-associated signaling.

**Results:**

Bioinformatic analysis identified TLR4 as a biologically relevant candidate hub gene potentially linking *P. gingivalis*-associated signaling and TIGIT-related immune regulation. Exposure to *P. gingivalis* significantly promoted tumor growth and increased the expression of TLR4, ERK1/2, CD155, and TIGIT in tumor tissues. Flow cytometric analysis revealed immune microenvironment remodeling characterized by increased proportions of CD4+ T cells, TIGIT-positive Tregs, and IL-10-positive cells, accompanied by reduced CD8+ T-cell infiltration and decreased frequencies of Ki67+CD8+ and CD44+CD8+ T cells. Treatment with the TLR4 inhibitor TAK-242 partially reversed these tumor-promoting, immunological, and molecular changes.

**Conclusion:**

Our findings suggest that exposure to *P. gingivalis* may promote OSCC progression by contributing to immune microenvironment remodeling. Increased expression of TLR4, ERK1/2, CD155, and TIGIT, together with the effects of TLR4 inhibition, supports the potential involvement of a TLR4-associated ERK/CD155/TIGIT signaling pathway in this process. However, the precise molecular mechanisms require further validation using genetic and functional approaches.

## 1 Introduction

Oral squamous cell carcinoma (OSCC) is one of the most common malignancies of the head and neck and remains associated with poor outcomes in advanced-stage disease despite improvements in multimodal therapy (1–4). Increasing evidence indicates that, in addition to genetic and environmental factors, the tumor microenvironment plays a critical role in OSCC progression, immune escape, and therapeutic resistance (5–7). The oral microbiota has emerged as an important regulator of oral carcinogenesis. Among oral pathogens, *Porphyromonas gingivalis*, a keystone periodontal bacterium, has been implicated in cancer development through its ability to promote chronic inflammation, tumor invasion, and immune dysregulation (8–10). Our previous studies further demonstrated that *P. gingivalis* contributes to the formation of a local immunosuppressive microenvironment in OSCC and that CCR6+ regulatory T cells are involved in this process (10, 11). However, the molecular mechanisms linking *P. gingivalis* exposure to tumor immune remodeling remain poorly understood.

Tumor immune escape is a hallmark of cancer progression, and immune checkpoint pathways play essential roles in this process (12). TIGIT is an inhibitory immune checkpoint receptor expressed on activated T cells, regulatory T cells (Tregs), and natural killer cells. Through interaction with its principal ligand CD155, TIGIT suppresses antitumor immune responses and contributes to the establishment of an immunosuppressive tumor microenvironment (13–15). Although both TIGIT and CD155 have been associated with poor prognosis in multiple malignancies, the upstream mechanisms regulating this pathway in OSCC remain unclear (13, 14).

Toll-like receptor 4 (TLR4), a key mediator of microbial recognition and innate immune signaling, has been implicated in inflammation-associated tumor progression (16–18). Activation of TLR4 can trigger downstream MAPK and NF-κB signaling pathways and regulate tumor-associated immune responses (19–21). Moreover, *P. gingivalis* has been reported to activate TLR4-related signaling in various cellular contexts (22, 23). Our previous work demonstrated that TGF-β1 upregulates CD155 through activation of ERK1/2 signaling in tongue squamous cell carcinoma (24), indicating that ERK1/2 acts upstream of CD155 and raising the possibility that other upstream stimuli, including TLR4 signaling, may converge on this axis. Therefore, the present study aimed to investigate whether a TLR4-associated ERK/CD155/TIGIT signaling pathway is involved in *P. gingivalis*-mediated immunosuppressive microenvironment formation during OSCC progression. Using bioinformatic analysis, animal experiments, immunohistochemistry, qRT-PCR and flow cytometry, we sought to evaluate the potential contribution of this pathway to microbial-associated immune regulation in OSCC.

## 2 Materials and methods

### 2.1 Bioinformatic analysis

Transcriptomic data and corresponding clinical information of patients with head and neck squamous cell carcinoma (HNSCC) were obtained from The Cancer Genome Atlas (TCGA) database (https://portal.gdc.cancer.gov/). According to the primary tumor site, a total of 236 oral squamous cell carcinoma (OSCC) cases were selected for subsequent analyses. Gene-expression data were normalized using log2(FPKM + 1) transformation prior to downstream analyses.

To identify genes associated with TIGIT, Pearson correlation analysis was performed using TCGA-OSCC transcriptomic data, and the top 1,000 genes ranked by correlation coefficient were selected as TIGIT-related genes. Genes associated with *Porphyromonas gingivalis* were retrieved from the GeneCards database (https://www.genecards.org), yielding 557 candidate genes. Overlapping genes between the TIGIT-related gene set and the *P. gingivalis*-associated gene set were identified using the jvenn online tool, resulting in 70 intersecting genes.

Protein–protein interaction (PPI) analysis of the overlapping genes was performed using the STRING database. The resulting PPI network was imported into Cytoscape (version 3.9.1) for visualization and hub-gene analysis. Candidate hub genes were identified using the Degree algorithm implemented in the cytoHubba plugin. Among the identified candidate hub genes, TLR4 was selected for subsequent investigation because of its established role in microbial recognition, innate immune signaling, and tumor-associated immune regulation.

### 2.2 Cell culture

Murine oral squamous cell carcinoma SCC7 cells were cultured in Dulbecco’s Modified Eagle Medium (DMEM; Gibco, USA) supplemented with 10% fetal bovine serum (FBS; Gibco, USA) and 1% penicillin–streptomycin solution under standard culture conditions (37°C, 5% CO_2_).

### 2.3 Preparation of *Porphyromonas gingivalis*

*Porphyromonas gingivalis* strain ATCC 33277 was cultured under anaerobic conditions according to standard protocols. Bacterial concentration was adjusted prior to experimental use. The purpose of bacterial administration in this study was to evaluate the biological consequences of *P. gingivalis* exposure on tumor progression rather than to establish a chronic infection model.

### 2.4 Animal model

To establish the tumor model, female C57BL/6 mice aged 6 to 8 weeks with qualified body weight were selected in this study, and the experimental design strictly followed the principle of randomization and control. Fifteen mice were randomly divided into three groups (control group, activation group, and TLR4 inhibitor intervention group) with 5 mice in each group: the control group was only inoculated with SCC7 tumor cells on day 0; the activation group was co-inoculated with a mixed suspension of SCC7 cells and *Porphyromonas gingivalis* (P.g) on day 0; the TLR4 inhibitor intervention group received daily intraperitoneal injection of TLR4 inhibitor for pretreatment 2 days before inoculation with the mixed suspension of SCC7 cells and P.g. The concentration of the prepared cell suspension was set as 2×10^6^ cells/mL, and the corresponding concentration of the bacterial suspension was 1×10^9^ CFU/mL. Experimental mice were anesthetized via intraperitoneal injection (2,2,2-tribromoethanol, 400 mg/kg, 20 μL/g), and after the mice in the control group were fully anesthetized, 100 μL of the cell suspension was immediately injected into the left buccal mucosa. After the induction of anesthesia, mice in the activation group received 100 μL of the mixed suspension of *Porphyromonas gingivalis* (P.g) and cells via the same route of left buccal mucosa injection. For the TLR4 inhibitor intervention group, daily intraperitoneal injection of TLR4 inhibitor (Resatorvid, TAK-242, at a dose of 3 mg/kg) was performed for 2 days before the experiment to complete pretreatment, and the subsequent procedure was consistent with that of the activation group (injection of 100 μL of the mixed suspension of P.g and cells into the left buccal mucosa). All experimental mice were euthanized by cervical dislocation 14 days after modeling, and tumor tissue samples were collected immediately. To assess tumor size, the long diameter (a, unit: mm) and short diameter (b, unit: mm) of tumors were accurately measured, and tumor volume was calculated using the formula V = 1/2 × a × b². For sample processing, partial tumor tissues were fixed with 4% paraformaldehyde solution for subsequent morphological observation, and the remaining partial tumor tissues were processed via combined digestion with collagenase IV and DNase I to prepare single-cell suspensions for flow cytometry or other analyses at the single-cell level. This study protocol has obtained strict approval from the Animal Experimental Ethics Review Committee of the First Affiliated Hospital of Xinjiang Medical University, with the approval number A240301-141, ensuring that the entire experimental process complies with ethical specifications.

### 2.5 Immunohistochemistry

Tumor tissues were fixed in 4% paraformaldehyde, embedded in paraffin, and sectioned at 4 μm thickness.

After deparaffinization, rehydration, antigen retrieval, and blocking, sections were incubated overnight at 4 °C with primary antibodies against:TLR4,ERK1/2,CD155,TIGIT.

After incubation with appropriate secondary antibodies, signals were visualized using diaminobenzidine (DAB) substrate and counterstained with hematoxylin.

### 2.6 Image acquisition and quantification

All images were acquired using identical microscope settings, exposure conditions, and magnifications.

For quantitative analysis, five randomly selected non-necrotic microscopic fields were analyzed from each section.

DAB-positive staining was quantified using the Color Deconvolution plugin in ImageJ software (NIH, USA).

The same threshold settings were applied to all images.

Mean optical density (MOD) values were calculated for each field and averaged for statistical analysis.

Representative images shown in figures were selected from sections demonstrating staining patterns consistent with quantitative results.

### 2.7 Flow cytometry

Fresh tumor tissues were harvested immediately after sacrifice and mechanically dissociated into single-cell suspensions. The cell suspensions were filtered through a 70-μm cell strainer and subjected to erythrocyte lysis. After washing with phosphate-buffered saline (PBS), cells were resuspended in staining buffer for flow cytometric analysis.

For surface-marker staining, cells were incubated with fluorochrome-conjugated antibodies against CD3, CD4, CD8, CD25, CD44, and TIGIT. For intracellular staining, cells were fixed and permeabilized according to the manufacturer's instructions and subsequently stained with antibodies against Foxp3, IL-10, TGF-β, IFN-γ, and Ki67.

The following immune-cell populations were analyzed:

CD4+ T cells

CD8+ T cells

Regulatory T cells (Tregs; CD4+CD25+Foxp3+)

TIGIT-positive Tregs

IL-10-positive immune cells

Ki67+CD8+ T cells

CD44+CD8+ T cells

IFN-γ+CD8+ T cells

TGF-β-producing immune cells

Ki67+ and CD44+ TIGIT+ / TIGIT- Treg subsets

Appropriate isotype controls and fluorescence-minus-one (FMO) controls were included for gating and threshold determination.

### 2.8 Gating strategy

Cell debris was excluded based on forward-scatter (FSC) and side-scatter (SSC) characteristics. Doublets were removed using FSC-A versus FSC-H gating. Lymphocytes were identified according to FSC/SSC parameters and subsequently gated on CD3+ T cells.

CD4+ and CD8+ T-cell populations were identified from the CD3+ compartment. Regulatory T cells were defined as CD4+CD25+Foxp3+ cells. TIGIT-positive Tregs were identified within the Treg population. Expression of IL-10 was analyzed in immune-cell populations according to the experimental design. Ki67 and CD44 expression was evaluated within CD8+ T cells and, separately, within TIGIT+ and TIGIT- Treg subsets to assess proliferation-associated and activation-associated characteristics. IFN-γ production was evaluated within CD8+ T cells, and TGF-β production was evaluated in tumor-infiltrating immune cells.

Positive populations were determined using FMO controls and corresponding negative controls. The same gating strategy and positivity thresholds were applied consistently across all experimental groups.

Flow cytometric data were acquired using a BD FACSCanto II flow cytometer (BD Biosciences, USA) and analyzed with FlowJo software (version X, Tree Star Inc., USA). Results were expressed as the percentage of positive cells within the corresponding immune-cell population.

### 2.9 Quantitative real-time PCR

Total RNA was extracted from tumor tissues using TRIzol reagent according to the manufacturer’s instructions.

RNA concentration and purity were assessed spectrophotometrically.

Complementary DNA (cDNA) was synthesized using a reverse transcription kit.

Quantitative PCR was performed using SYBR Green Master Mix on a real-time PCR system.

The following genes were analyzed:TLR4,ERK1/2,CD155,TIGIT.

GAPDH served as the internal control.

Relative gene expression was calculated using the 2^-ΔΔCt method.

### 2.10 Statistical analysis

All data are presented as mean ± standard deviation (SD).

Statistical analyses were performed using GraphPad Prism software.

Comparisons between two groups were conducted using Student’s t-test.

Comparisons among multiple groups were performed using one-way analysis of variance (ANOVA) followed by appropriate *post hoc* tests.

A two-sided P value < 0.05 was considered statistically significant.

## 3 Results

### 3.1 Bioinformatic analysis identified TLR4 as a candidate hub gene potentially linking *P. gingivalis*-associated signaling and TIGIT-related immune regulation

To explore the potential molecular mechanism underlying *P. gingivalis*-mediated immune regulation in OSCC, *P. gingivalis*-related genes were retrieved from the GeneCards database, and TIGIT-correlated genes were identified using TCGA-HNSC transcriptomic data. Venn analysis was then performed to determine the overlapping genes between these two gene sets (**Figure 1A**). The overlapping genes were further subjected to STRING-based protein–protein interaction (PPI) network analysis and Cytoscape visualization (**Figure 1B**). Using cytoHubba analysis, several candidate hub genes were identified within the interaction network (**Figure 1C**). Among them, TLR4 attracted particular attention because of its established role in bacterial recognition and innate immune signaling. Moreover, as a pattern-recognition receptor located upstream of the immune signaling cascade, TLR4 was selected for subsequent validation. Correlation analysis further showed a positive association between TLR4 and TIGIT expression (**Figure 1D**), suggesting that TLR4 may serve as a potential molecular link between *P. gingivalis*-related stimulation and TIGIT-associated immune regulation in OSCC.

**Figure 1:**
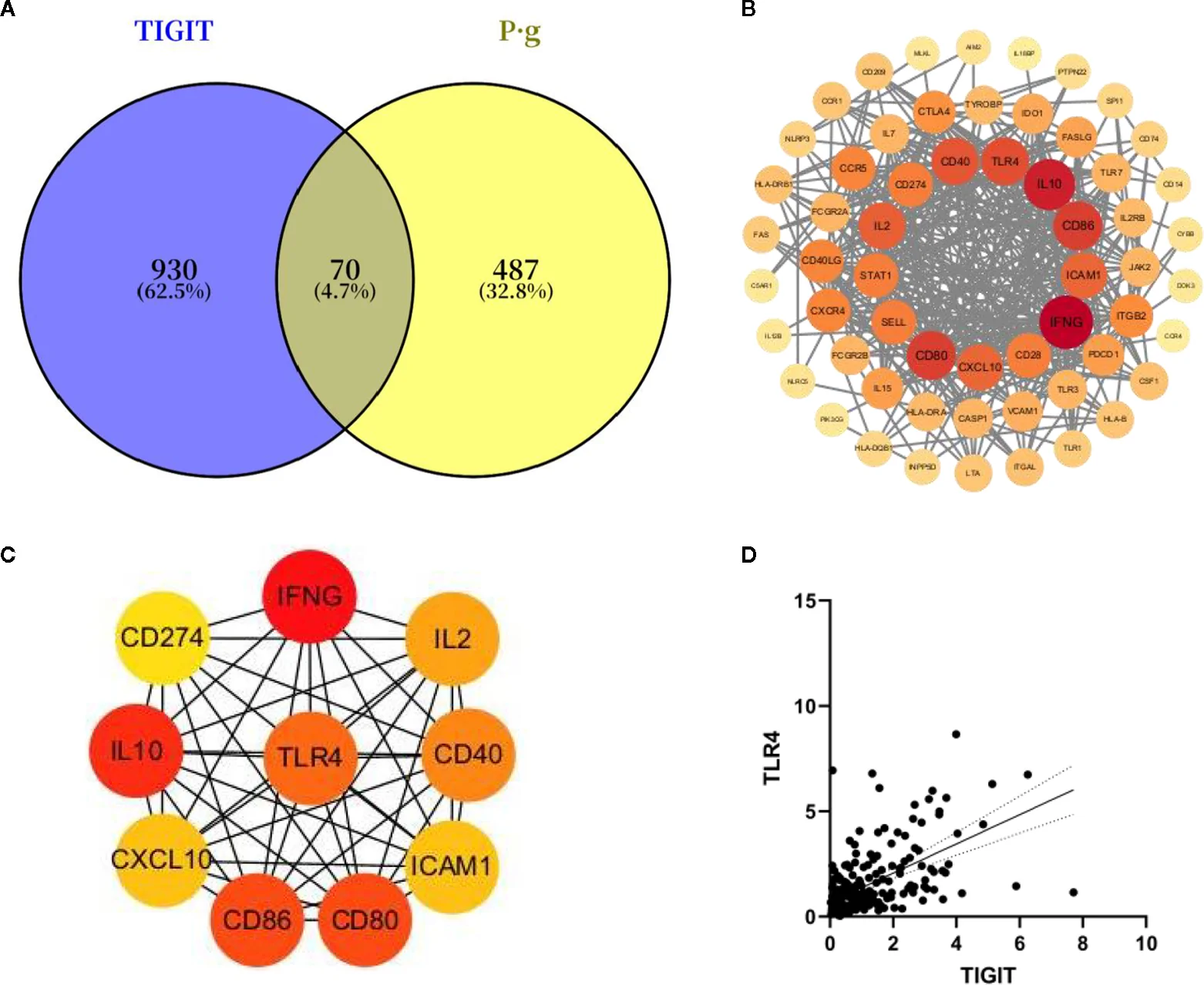
Identification of a candidate hub gene. **(A)** Venn diagram showing the overlap between (P) gingivalis-associated genes and TIGIT-related genes identified from TCGA-HNSC data. **(B)** Protein–protein interaction (PPI) network constructed using the STRING database. **(C)** Candidate hub genes identified using Cytoscape and cytoHubba analysis. **(D)** Correlation analysis of TLR4 and TIGIT.

### 3.2 *P. gingivalis* promoted tumor growth *in vivo*, which was partially attenuated by TLR4 inhibition

The experimental design and grouping are summarized in **Figure 2A**, and HE staining of tumor tissues from the three groups is shown in **Figure 2B**. As shown in **Figures 2C, D**, exposure to *P. gingivalis* significantly increased tumor volume and tumor weight compared with the SCC7 control group (P < 0.05). Administration of the TLR4 inhibitor TAK-242 partially reversed these effects, resulting in significantly reduced tumor growth compared with the SCC7 + *P. gingivalis* group (P < 0.05).These findings suggest that TLR4-associated signaling may contribute to *P. gingivalis*-mediated tumor-promoting effects in OSCC.

**Figure 2:**
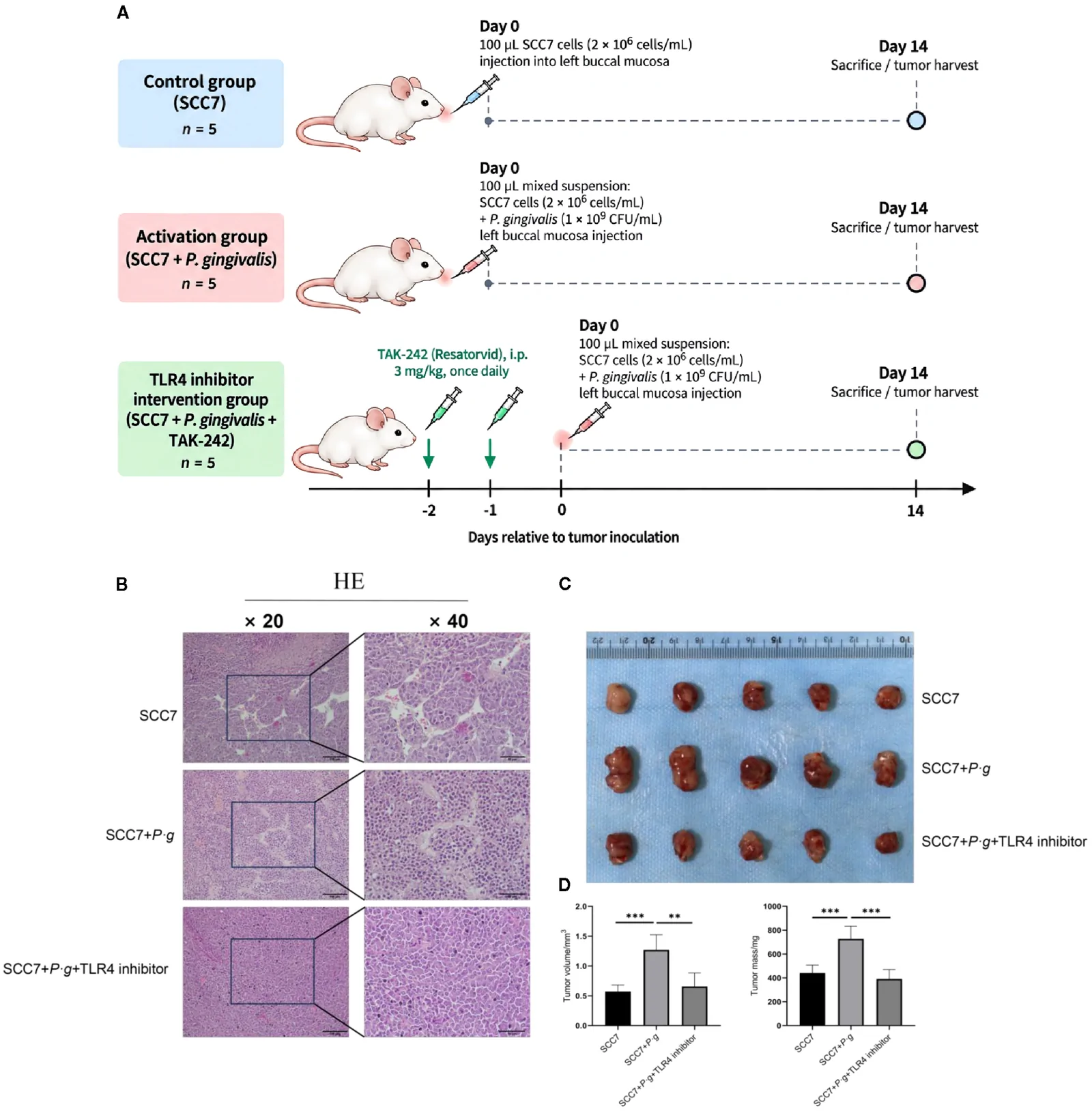
**(A)** Schematic diagram of model establishment and grouping; **(B)** HE staining; **(C)** Gross images of tumors **(D)** Statistical analysis of tumor volume and tumor weight. **P < 0.01, ***P < 0.001.

### 3.3 *P. gingivalis* enhances TLR4, ERK1/2, CD155 and TIGIT expression in OSCC tissues, which is partially reversed by TLR4 inhibition

To further evaluate whether *P. gingivalis* was associated with activation of the proposed TLR4/ERK1/2/CD155/TIGIT-related pathway *in vivo*, immunohistochemistry and qRT-PCR were performed on tumor tissues from the control, *P. gingivalis*-treated, and TAK-242 intervention groups. Immunohistochemical staining showed that the expression levels of TLR4, ERK1/2, CD155 and TIGIT were increased in tumor tissues after *P. gingivalis* treatment compared with the control group (**Figures 3A–D**). In contrast, pretreatment with the TLR4 inhibitor TAK-242 partially reduced the *P. gingivalis*-induced upregulation of these markers as confirmed by quantitative analysis of mean optical density (**Figures 3E–H**).

**Figure 3:**
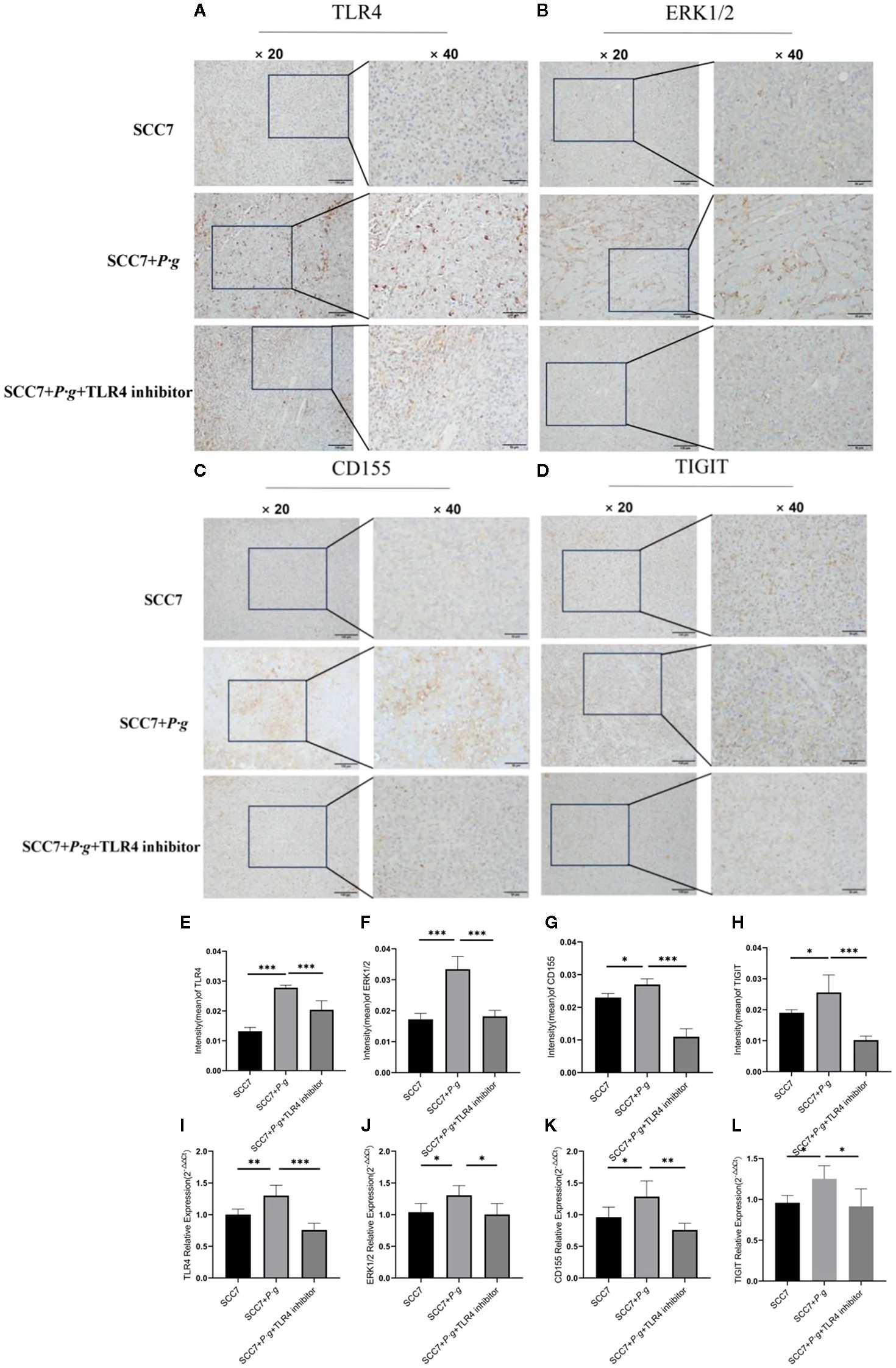
**(A–D)** Representative immunohistochemical staining images of TLR4, ERK1/2, CD155 and TIGIT in tumor tissues from the control, *P. gingivalis*-treated and TAK-242 intervention groups. **(E–H)** Quantitative analysis of immunohistochemical staining for TLR4, ERK1/2, CD155 and TIGIT based on mean optical density. **(I–L)** Relative mRNA expression levels of TLR4, ERK1/2, CD155 and TIGIT detected by qRT-PCR and normalized to GAPDH.Data are presented as mean ± SD. *P < 0.05, **P < 0.01, ***P < 0.001.

Consistently, qRT-PCR analysis showed a similar trend at the mRNA level. Compared with the control group, *P. gingivalis* treatment increased the relative mRNA expression of TLR4, ERK1/2, CD155 and TIGIT, whereas TAK-242 intervention partially reversed these changes (**Figures 3I–L**). These findings suggest that *P. gingivalis*-associated modulation of the TLR4/ERK1/2/CD155/TIGIT-related pathway may contribute to the formation of an immunosuppressive tumor microenvironment in OSCC.

### 3.4 TLR4 blockade suppresses *P. gingivalis*-induced TIGIT expression in tumor-infiltrating lymphocytes

Flow cytometry analysis showed that *P. gingivalis* significantly increased the proportions of TIGIT-positive natural killer cells, CD8^+^ T cells, and CD4^+^ T cells within the tumor microenvironment (**Figures 4A–C**). Among these immune-cell subsets, CD4^+^ T cells exhibited the most prominent TIGIT expression (**Figure 4D**), suggesting that CD4^+^ T cell-mediated immunoregulation may play an important role in *P. gingivalis*-induced immunosuppressive responses in OSCC. Following TLR4 inhibition, the proportions of TIGIT-positive cells were significantly reduced across these immune-cell subsets (Figures 4A-C), indicating that TLR4 signaling may be involved in *P. gingivalis*-associated activation of TIGIT-related immune checkpoint responses.

**Figure 4:**
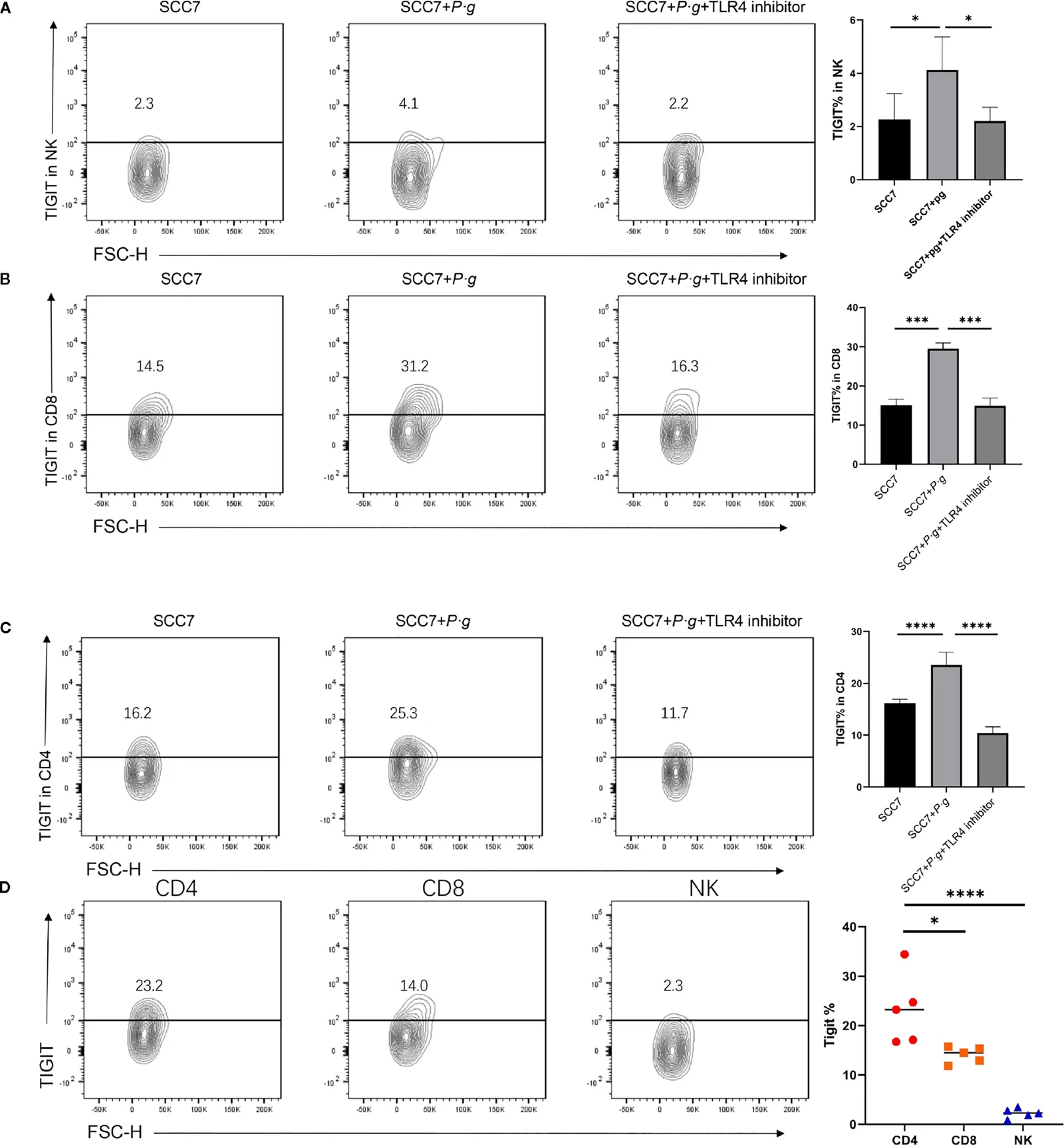
TLR4 inhibition reduces *P. gingivalis*-associated TIGIT expression in tumor-infiltrating immune-cell subsets. **(A–C)** Quantitative analysis of the proportions of TIGIT-positive NK cells, CD8^+^ T cells, and CD4^+^ T cells in the SCC7, SCC7 + *P. gingivalis*, and SCC7 + *P. gingivalis* + TAK-242 groups. **(D)** Distribution of TIGIT expression among tumor-infiltrating immune-cell subsets, showing that TIGIT expression was most prominent in CD4^+^ T cells. Data are presented as mean ± SD. *P < 0.05, ***P < 0.001, ****P < 0.0001.

### 3.5 TIGIT is preferentially expressed on Treg cells, and *P. gingivalis* increases TIGIT^+^ Treg cells through TLR4 signaling

TIGIT expression was markedly higher in tumor-infiltrating Treg cells than in Teff cells (**Figure 5A**). Flow cytometric analysis showed that the overall proportion of Treg cells did not differ significantly between the SCC7 and SCC7 + *P. gingivalis* groups, whereas TAK-242 intervention significantly reduced Treg infiltration (**Figure 5B**). In contrast, *P. gingivalis* markedly increased the proportion of TIGIT^+^ Treg cells, and this increase was reversed by TAK-242 (**Figure 5C**). These findings suggest that TLR4 signaling may participate in *P. gingivalis*-associated immunosuppressive responses and contribute to the development of an immunosuppressive tumor microenvironment in OSCC.

**Figure 5:**
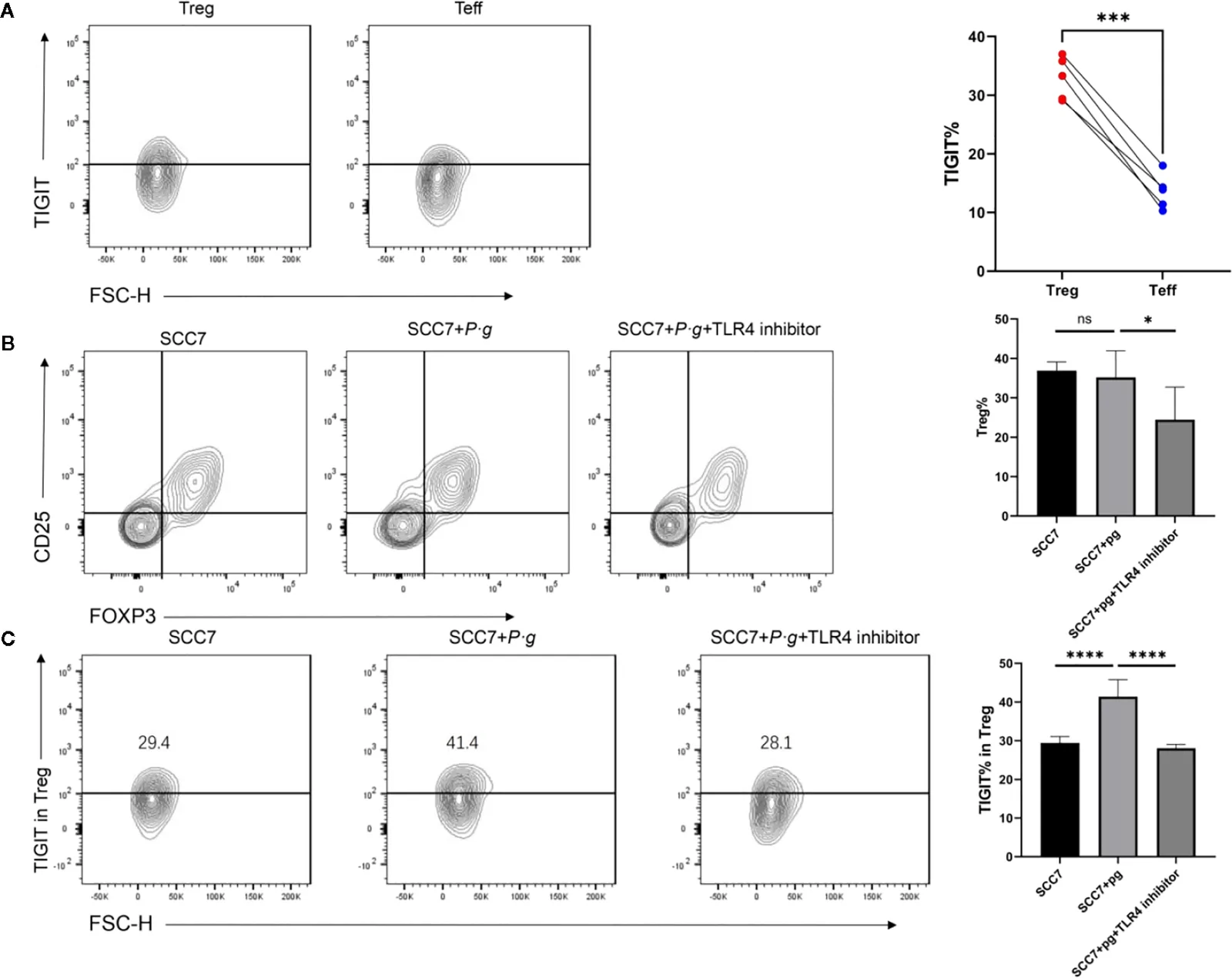
Proportions of Treg and TIGIT^+^ Treg cell subpopulations in the OSCC tumor microenvironment. **(A)** Representative flow cytometry plots and quantitative comparison of TIGIT expression between tumor-infiltrating Treg and Teff cells. **(B)** Representative CD25/FOXP3 plots and quantitative analysis of the proportion of Treg cells in the SCC7, SCC7 + *P. gingivalis*, and SCC7 + *P. gingivalis* + TAK-242 groups. **(C)** Representative plots and quantitative analysis of the proportion of TIGIT^+^ Treg cells in the same three groups. Data are presented as mean ± SD. ns, not significant; *P < 0.05, ***P < 0.001, ****P < 0.0001.

### 3.6 TIGIT⁺ Treg cells exhibit enhanced proliferative and activation-associated phenotypes, and *P. gingivalis* increases IL-10 production

Ki67 and CD44 expression was first compared between tumor-infiltrating Treg and Teff cells. Both markers were expressed at higher levels in Treg cells, which were therefore selected for further analysis and subdivided into TIGIT^+^ and TIGIT⁻ subsets. Compared with TIGIT⁻ Treg cells, TIGIT^+^ Treg cells showed a significantly higher proportion of Ki67^+^ cells (**Figure 6A**) and a significantly higher proportion of CD44^+^ cells (**Figure 6B**), consistent with an activated, effector-like Treg phenotype. In addition, *P. gingivalis* exposure increased the proportion of IL-10-producing immune cells, and this increase was partially reduced following TAK-242 intervention (**Figure 6C**), whereas TGF-β expression did not differ significantly among the three groups (**Figure 6D**). These results indicate that TIGIT^+^ Treg cells constitute a highly proliferative and activated Treg subpopulation with an enhanced immunosuppressive phenotype, and that the *P. gingivalis*-associated immunosuppressive milieu in this model is characterized predominantly by increased IL-10 production.

**Figure 6:**
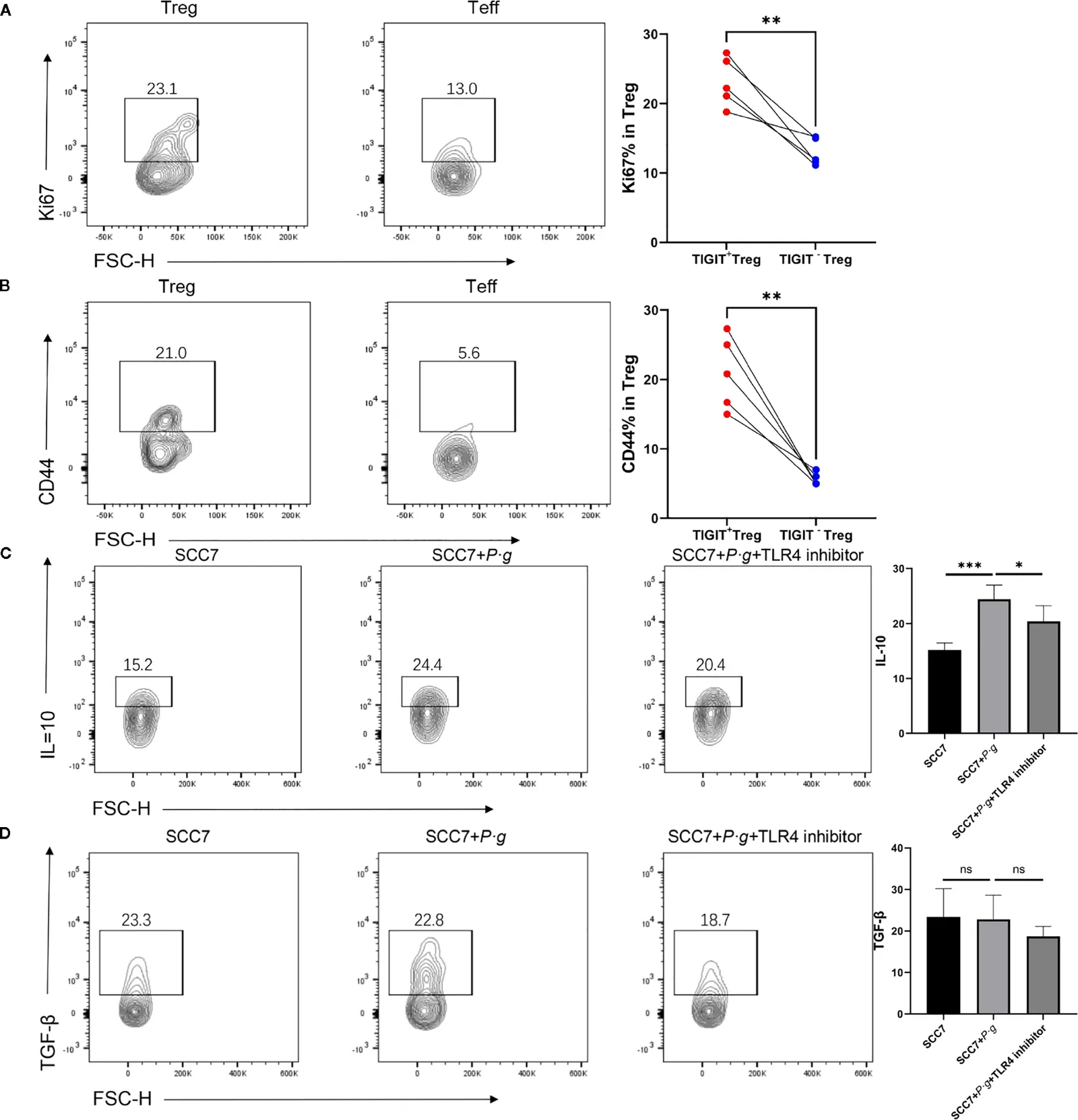
Phenotype of TIGIT^+^ Treg cells and immunosuppressive cytokine expression in the OSCC tumor microenvironment. **(A)** Representative flow cytometry plots and quantitative analysis of Ki67 expression in TIGIT^+^ versus TIGIT⁻ Treg cells. **(B)** Representative plots and quantitative analysis of CD44 expression in TIGIT^+^ versus TIGIT⁻ Treg cells. **(C)** Representative plots and quantitative analysis of IL-10-producing immune cells in the SCC7, SCC7 + *P. gingivalis*, and SCC7 + *P. gingivalis* + TAK-242 groups. (D) Representative plots and quantitative analysis of TGF-β-producing immune cells in the same three groups. Data are presented as mean ± SD. ns, not significant; *P < 0.05, **P < 0.01, ***P < 0.001.

### 3.7 TLR4 inhibition partially restores the activation- and proliferation-associated phenotype of CD8^+^ T cells following *P. gingivalis* exposure

Although *P. gingivalis* did not significantly alter the overall proportion of CD8^+^ T cells, it reduced the proportions of Ki67^+^CD8^+^ T cells and CD44^+^CD8^+^ T cells (**Figures 7A, B**), indicating decreased proliferation- and activation-associated phenotypes (P < 0.05). In addition, the proportion of IFN-γ-producing CD8⁺ T cells was significantly reduced following *P. gingivalis* exposure, and this reduction was not significantly restored by TAK-242 (**Figure 7C**). These findings suggest that *P. gingivalis* may impair the activation- and proliferation-related characteristics of tumor-infiltrating CD8^+^ T cells, whereas TLR4 inhibition partially reverses these alterations.

**Figure 7:**
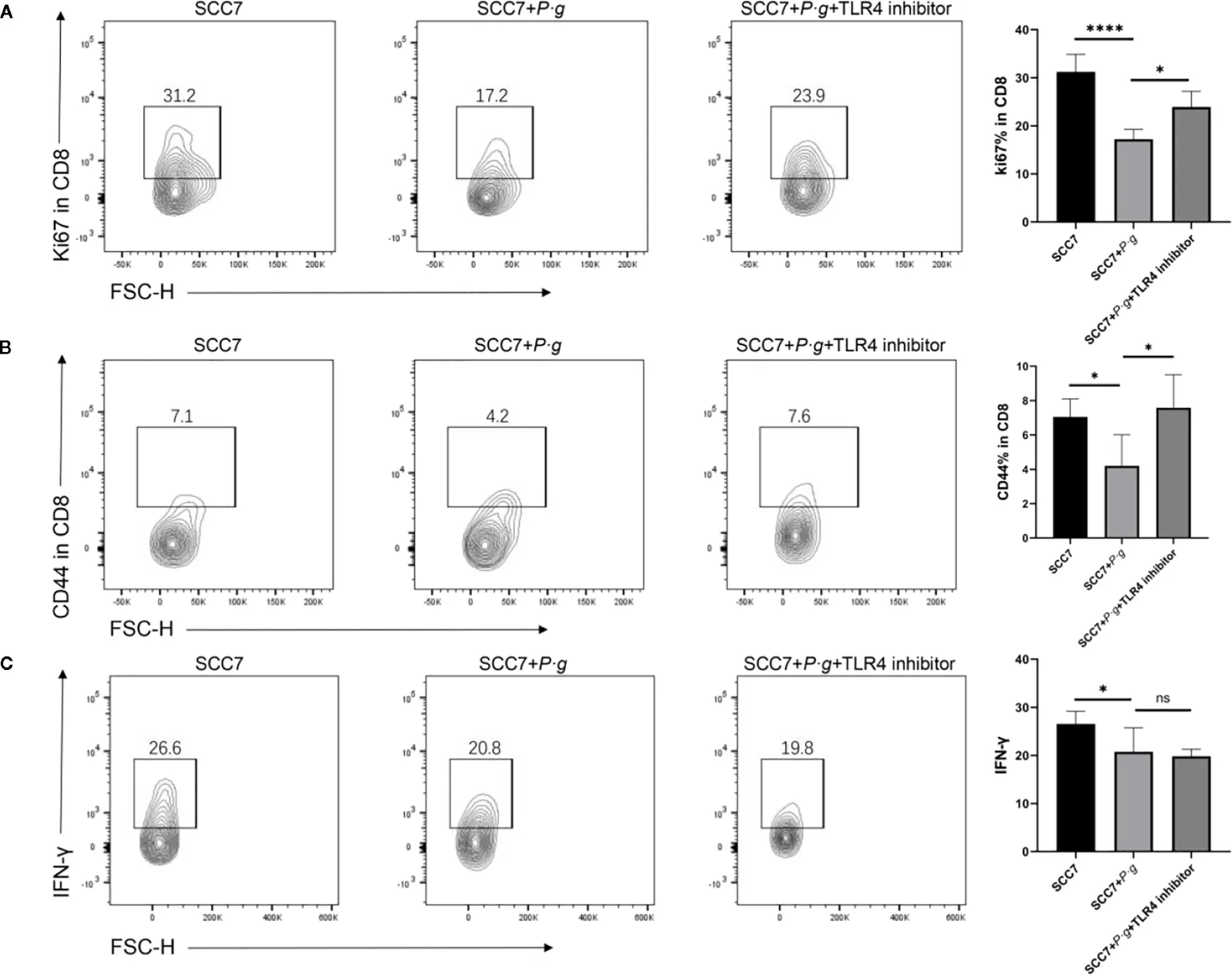
TLR4 inhibition partially restores the activation- and proliferation-associated phenotype of CD8^+^ T cells following *P. gingivalis* exposure. (A) Representative flow cytometry plots and quantitative analysis of Ki67^+^CD8^+^ T cells in the SCC7, SCC7 + *P. gingivalis*, and SCC7 + *P. gingivalis* + TAK-242 groups. (B) Representative plots and quantitative analysis of CD44^+^CD8^+^ T cells in the same three groups. (C) Representative plots and quantitative analysis of IFN-γ-producing CD8^+^ T cells in the same three groups. Data are presented as mean ± SD. ns, not significant; *P < 0.05, ****P < 0.0001.

## 4 Discussion

Increasing evidence indicates that oral microbial dysbiosis contributes to the initiation and progression of oral squamous cell carcinoma (OSCC) (8, 9). Among oral pathogens, *Porphyromonas gingivalis* has attracted particular attention because of its ability to modulate inflammatory responses, alter epithelial-cell behavior, and influence host immunity (9–11). However, the molecular mechanisms linking *P. gingivalis* exposure to immune microenvironment remodeling in OSCC remain incompletely understood.

In the present study, we combined bioinformatic analysis, animal experiments, immunohistochemistry, flow cytometry, and qRT-PCR to investigate the potential involvement of TLR4-associated signaling in *P. gingivalis*-mediated immune regulation. Our findings demonstrated that exposure to *P. gingivalis* was associated with accelerated tumor growth, increased expression of TLR4, ERK1/2, CD155, and TIGIT, as well as the development of an immunosuppressive phenotype within the tumor microenvironment. Furthermore, pharmacological inhibition of TLR4 partially reversed these changes.

TLR4 is a well-characterized pattern-recognition receptor involved in innate immune responses against microbial pathogens (16, 17). Previous studies have demonstrated that *P. gingivalis*-derived virulence factors can activate TLR4-dependent signaling pathways, thereby inducing inflammatory responses and promoting tumor-associated biological processes (22, 23). Consistent with these reports, our bioinformatic analysis identified TLR4 as a biologically relevant candidate hub gene potentially linking *P. gingivalis*-associated signaling and TIGIT-related immune regulation. Importantly, TLR4 inhibition significantly attenuated the tumor-promoting effects observed following *P. gingivalis* exposure, suggesting that TLR4-associated signaling may contribute to this process.

Accumulating evidence suggests that TLR4 signaling is closely associated with tumor progression and immune escape in various malignancies (17–20). In OSCC, elevated TLR4 expression has been correlated with aggressive clinicopathological characteristics and impaired antitumor immune responses (25–27). Furthermore, activation of TLR4 signaling has been reported to induce immunosuppressive cytokine production, apoptosis resistance, and immune evasion in tumor cells (19, 21, 27). Notably, TLR4 expression has been reported to show an inverse relationship with PD-L1 in the inflammatory microenvironment of OSCC (28), indicating that TLR4 may influence several immune-regulatory axes in addition to the CD155/TIGIT pathway examined here. Therefore, TLR4 may represent an important molecular bridge connecting microbial stimulation and tumor immune regulation.

One noteworthy observation in the present study was the coordinated increase in TLR4, ERK1/2, CD155, and TIGIT expression. Previous studies have shown that activation of ERK/MAPK signaling can regulate tumor-cell proliferation, migration, and immune-related gene expression (23). Our previous work demonstrated that TGF-β1-induced ERK1/2 activation upregulates CD155 in tongue squamous cell carcinoma (24). CD155 is the principal ligand of TIGIT and plays a crucial role in the establishment of immunosuppressive tumor microenvironments (13, 14). Therefore, the observed expression pattern may indicate a potential relationship among TLR4 activation, ERK1/2 signaling, CD155 upregulation, and TIGIT-associated immune regulation. However, the present data demonstrate association rather than direct causality. Additional mechanistic studies are required to determine whether these molecules function within a linear signaling cascade.

Flow cytometric analysis revealed substantial alterations in the tumor immune microenvironment following *P. gingivalis* exposure. Increased proportions of TIGIT-positive Tregs and IL-10-producing cells were observed together with reduced CD8+ T-cell infiltration and diminished expression of activation-associated markers, including Ki67 and CD44 (11–14).

These findings are consistent with our previous work and with other reports demonstrating that *P. gingivalis* promotes the establishment of an immunosuppressive tumor microenvironment through recruitment and activation of regulatory immune-cell populations (10, 11). Tregs are recognized as major mediators of tumor immune escape, whereas TIGIT signaling contributes to suppression of antitumor immune responses and enhancement of regulatory T-cell function (13–15). In addition, increased IL-10 production has been associated with inhibition of effector T-cell activity and maintenance of an immunosuppressive microenvironment (12, 21).

Collectively, the immune alterations observed in the present study support the notion that *P. gingivalis* exposure contributes to immune microenvironment remodeling characterized by enhanced immunosuppressive activity and reduced antitumor immune responses.

In addition, *P. gingivalis* exposure was associated with reduced proportions of Ki67+CD8+ T cells and CD44+CD8+ T cells, suggesting impaired activation-associated and proliferative characteristics of antitumor T cells. These observations further support the presence of an immunosuppressive immune landscape within the tumor microenvironment. However, because direct functional assays evaluating T-cell proliferation, cytokine secretion, and cytotoxic activity were not performed, the present findings should be interpreted as evidence of an immunosuppression-associated phenotype rather than definitive proof of functional T-cell exhaustion.

The effects of TAK-242 observed in the present study provide additional support for the involvement of TLR4-associated signaling. Nevertheless, *P. gingivalis* is known to activate multiple pattern-recognition receptors, including TLR2, TLR4, TLR9, and CD14 (20, 22). Therefore, the contribution of parallel signaling pathways cannot be excluded. The observed effects following TAK-242 administration suggest the importance of TLR4 signaling but do not establish TLR4 as the sole mediator of *P. gingivalis*-induced immune modulation.

Collectively, our findings suggest that immune microenvironment remodeling may represent an important mechanism by which *P. gingivalis* contributes to OSCC progression. Understanding the interactions among microbial stimulation, innate immune signaling, and immune checkpoint regulation may provide novel opportunities for the development of targeted therapeutic strategies in OSCC.

## 5 Limitations

Several limitations should be acknowledged.

First, the bioinformatic analysis performed in this study was primarily intended to identify biologically plausible candidate genes for downstream investigation. Although TLR4 emerged as a candidate hub gene, bioinformatic analysis alone cannot establish causal relationships.

Second, the TCGA-based transcriptomic analysis utilized bulk RNA-sequencing data. Because bulk RNA-seq cannot distinguish tumor-cell-derived signals from immune-cell-derived signals, differences in immune-cell infiltration may contribute to the observed gene-expression patterns. Future studies using immune-cell deconvolution algorithms, single-cell RNA sequencing, or spatial transcriptomics may provide more precise cellular resolution.

Third, the animal model employed in this study was designed to evaluate the biological consequences of *P. gingivalis* exposure rather than chronic bacterial colonization. We did not directly assess bacterial persistence, viability, or localization within tumor tissues during the experimental period. Therefore, our findings should be interpreted as the effects of *P. gingivalis* exposure rather than proof of sustained infection.

Fourth, mechanistic conclusions were primarily based on pharmacological inhibition using TAK-242. Although TAK-242 significantly attenuated the observed effects, genetic approaches such as TLR4 knockdown, TLR4 knockout, or cell-type-specific manipulation would provide stronger evidence regarding causality.

Fifth, the present study evaluated TIGIT and IL-10 expression but did not include additional immune-checkpoint markers such as PD-1, TIM-3, or LAG-3, nor did it assess T-cell proliferation, cytotoxicity, or cytokine secretion. Therefore, definitive characterization of functional T-cell exhaustion was beyond the scope of the current study.

Finally, although immunohistochemistry and qRT-PCR demonstrated coordinated changes in TLR4, ERK1/2, CD155, and TIGIT expression, direct molecular interactions among these signaling components were not investigated. Future studies incorporating genetic manipulation, pathway-specific inhibitors, and functional assays will be necessary to establish the precise molecular mechanisms involved.

## 6 Conclusion

In summary, our findings suggest that exposure to *Porphyromonas gingivalis* may promote OSCC progression and contribute to the formation of an immunosuppressive tumor microenvironment. Increased expression of TLR4, ERK1/2, CD155, and TIGIT, together with the effects of TLR4 inhibition, support the potential involvement of a TLR4-associated ERK/CD155/TIGIT pathway in this process. However, additional genetic and functional studies are required to further validate the underlying mechanisms.

## Data Availability

All data generated or analysed during this study are included in this article. The transcriptomic and clinical data analysed in this study are publicly available from The Cancer Genome Atlas (https://portal.gdc.cancer.gov/) and the GeneCards database (https://www.genecards.org). Further inquiries can be directed to the corresponding author.
