# Correction: Potential involvement of a TLR4-associated CD155/TIGIT pathway in *Porphyromonas gingivalis*-induced immunosuppressive microenvironment formation in oral squamous cell carcinoma

**DOI:** 10.3389/fonc.2026.2020462

**Published:** 2026-11-06

**Authors:** Xiao Tian, Liming Xu, Yulian Fu, Qiang Qiang, Xue Cao, Jie Wang, Langqing Liu, Bin Ling

**Affiliations:** 1 Department of Oral and Maxillofacial Oncology Surgery, First Affiliated Hospital of Xinjiang Medical University, Urumqi, China; 2 Affiliated Stomatology Hospital, Institute of Stomatology, Xinjiang Uygur Autonomous Region, Urumqi, China

**Keywords:** CD155, oral squamous cell carcinoma, *Porphyromonas gingivalis*, TIGIT, TLR4, tumor immune microenvironment

Affiliation 1, “Department of Oral and Maxillofacial Oncology Surgery, First Affiliated Hospital of Xinjiang Medical University, Urumqi, China”, was erroneously omitted for author “Bin Ling”. This affiliation has now been added for author “Bin Ling”.

The original version of this article has been updated.

